# Comparative Sequence, Structure and Redox Analyses of *Klebsiella pneumoniae* DsbA Show That Anti-Virulence Target DsbA Enzymes Fall into Distinct Classes

**DOI:** 10.1371/journal.pone.0080210

**Published:** 2013-11-14

**Authors:** Fabian Kurth, Kieran Rimmer, Lakshmanane Premkumar, Biswaranjan Mohanty, Wilko Duprez, Maria A. Halili, Stephen R. Shouldice, Begoña Heras, David P. Fairlie, Martin J. Scanlon, Jennifer L. Martin

**Affiliations:** 1 Division of Chemistry and Structural Biology, Institute for Molecular Bioscience, The University of Queensland, Brisbane, Queensland, Australia; 2 Faculty of Pharmacy and Pharmaceutical Sciences, Medicinal Chemistry, Monash Institute of Pharmaceutical Sciences, Monash University, Parkville, Victoria, Australia; 3 ARC Centre of Excellence for Coherent X-ray Science, Monash University, Parkville, Victoria, Australia; University of South Florida College of Medicine, United States of America

## Abstract

Bacterial DsbA enzymes catalyze oxidative folding of virulence factors, and have been identified as targets for antivirulence drugs. However, DsbA enzymes characterized to date exhibit a wide spectrum of redox properties and divergent structural features compared to the prototypical DsbA enzyme of *Escherichia coli* DsbA (EcDsbA). Nonetheless, sequence analysis shows that DsbAs are more highly conserved than their known substrate virulence factors, highlighting the potential to inhibit virulence across a range of organisms by targeting DsbA. For example, *Salmonella enterica typhimurium* (SeDsbA, 86 % sequence identity to EcDsbA) shares almost identical structural, surface and redox properties. Using comparative sequence and structure analysis we predicted that five other bacterial DsbAs would share these properties. To confirm this, we characterized *Klebsiella pneumoniae* DsbA (KpDsbA, 81 % identity to EcDsbA). As expected, the redox properties, structure and surface features (from crystal and NMR data) of KpDsbA were almost identical to those of EcDsbA and SeDsbA. Moreover, KpDsbA and EcDsbA bind peptides derived from their respective DsbBs with almost equal affinity, supporting the notion that compounds designed to inhibit EcDsbA will also inhibit KpDsbA. Taken together, our data show that DsbAs fall into different classes; that DsbAs within a class may be predicted by sequence analysis of binding loops; that DsbAs within a class are able to complement one another *in vivo* and that compounds designed to inhibit EcDsbA are likely to inhibit DsbAs within the same class.

## Introduction

Antibiotic resistance has increased dramatically over the last decade and the consequent lack of treatment options poses a major threat for public health [1]. One approach to develop new chemical classes of antibacterials is to target virulence factors that cause disease in antibiotic resistant organisms [2]. Most pathogenic *Enterobacteriaceae* encode an oxidative folding pathway essential for virulence factor production [2-5]. Typically, the oxidative folding machinery includes a soluble thioredoxin-fold protein, DsbA, and an integral membrane protein partner, DsbB [6-8]. The disulfide form of DsbA is highly oxidizing and donates its disulfide bond to unfolded substrate proteins [9], leaving DsbA in the inactive reduced form. The inner membrane protein DsbB, in concert with its cofactor ubiquinone, interacts with reduced DsbA to oxidize the active site cysteines and convert DsbA to its functionally competent disulfide form [10]. Inhibition of the interaction between DsbA and substrate proteins or between DsbA and its partner DsbB could constitute a means of blocking virulence factor formation and thereby of inhibiting virulence of bacterial pathogens. Supporting this notion, deletion of DsbA homologues in pathogenic organisms results in diminished virulence in infection models [2,11] and deletion of *dsbA* or *dsbB* in uropathogenic *E. coli* (UPEC) severely attenuated its ability to colonize the bladder [11,12].

The characteristic properties of EcDsbA include: an active site CPHC motif that forms a destabilizing disulfide (T_m_ reduced EcDsbA 350 K; T_m_ oxidized EcDsbA 342 K) [13]; the more N-terminal of the two cysteines is nucleophilic and highly acidic, pK_a_ 3.3 (usual value for a cysteine is 8-9) [9]; and EcDsbA is highly oxidizing (redox potential -122 mV) [9]. The past 5 years has seen the characterization of DsbA enzymes from many other bacteria including DsbAs with varying degrees of sequence identity to EcDsbA such as *Neisseria meningitidis* DsbA1 (NmDsbA1, 23% identity), *Pseudomonas aeruginosa* DsbA (PaDsbA, 30%) and *Vibrio cholerae* DsbA (VcDsbA, or TcpG, 40%). These DsbAs share a similar structural fold with EcDsbA though their surface properties vary [14] and they exhibit a wide range of redox properties (Table **1**). Importantly, the EcDsbA hydrophobic groove that interacts with its essential partner EcDsbB is considerably truncated in NmDsbA1, PaDsbA and VcDsbA [15-17]. This modification and other surface changes in these DsbAs indicate that they fall into a separate class, distinct from EcDsbA, and that inhibitors designed against EcDsbA may not inhibit members of this class of DsbA. Conversely, DsbAs closely related to EcDsbA should be susceptible to the same mode of chemical inhibition. 

**Table 1:** Comparison of structures and redox properties of DsbAs.

	**Seq id to EcDsbA**	**RMSD (Å)**	**RMSD #Cα**	**E°’ (mV)**	**pK_a_ “Cys30”**	**T_m_ (K) (red/ox)**
Other DsbAs ^a^	10 - 40 %	1.3 - 2.9	122 - 167	-80/-163	3.0 - 5.1	337-357 / 331-341
EcDsbA^b^	100 %	0.6	176	-122	3.3	350 / 341
SeDsbA^c^	86 %	0.9	176	-126	3.3	351 / 343
KpDsbA	81 %	0.8	176	-116	3.2	347 / 335
VcDsbA^d^	40 %	1.8	168	- 116	5.1	357 / 346
NmDsbA1^e^	23 %	2.6	163	- 80	3.0	348 / 333

a [14] , redox potential range for NmDsbA1 (-80 , WpDsbA (- 163); pK_a_ range, NmDsbA1 (3.0), VcDsbA (5.1); T_m_ oxidised (min) NmDsbA3, (max) VcDsbA and reduced (min) NmDsbA3, (max) VcDsbA.

b [6] [14],, RMSD of EcDsbA derived from the overlay of molecules A and B from the asymmetric unit in 1FVK.

c [43] and [14]

d [54]

e [51]

Here we tested how close the sequence relationship must be to produce similar redox properties and binding interactions. We investigated two well-characterised DsbAs sharing 86% sequence identity, from *E. coli* K-12 strain (EcDsbA) and *S. enterica Typhimurium* DsbA strain SL1344 (SeDsbA), by applying comparative structural, sequence and redox analyses to identify properties conserved across these two enzymes. The results allow us to place DsbAs of five other Gram-negative bacteria *Enterobacteriaceae*, namely *Shigella flexneri* 8401 (SfDsbA, 100% sequence identity to EcDsbA), *Enterobacter cloacae* SCF-1 (EnDsbA, 84%), *Citrobacter koseri* ATCC BAA-895 (CkDsbA, 84%), *Cronobacter sakazakii* SP291 (CsDsbA, 82%) and *K. pneumonia* 342 (KpDsbA, 81%) into the same DsbA cluster as SeDsbA and EcDsbA. To assess whether the redox and structural properties are maintained in this DsbA group we focused on KpDsbA, which shares the lowest sequence identity with EcDsbA. We determined the high resolution crystal structure of reduced KpDsbA and the NMR solution structure of oxidized KpDsbA, and we measured the redox properties of this enzyme. As expected, the redox properties, surface characteristics and binding properties of KpDsbA are similar to those of EcDsbA suggesting that inhibitors developed against EcDsbA are likely to also be effective against other members of this DsbA subclass. 

## Materials and Methods

### Protein production

Codon-optimized *K. pneumoniae dsbA* (GenBank® accession number ACI08793), lacking the sequence coding for the predicted signal sequence (19 aa), was cloned into a modified pMCSG7 (Midwest Center for Structural Genomics) vector compatible with ligation-independent cloning. This modified vector encoded a leader sequence consisting of an N-terminal His_6_-tag followed by a linker containing the tobacco-etch virus protease (TEV) recognition sequence. KpDsbA was expressed in BL21(DE3)pLys cells using autoinduction medium [18] and purified with Talon® resin (Clontech, Australia). The His_6_-tag was removed by TEV protease, leaving the engineered KpDsbA with two additional amino acids (S–1 and N0) at the N-terminus. A final size-exclusion chromatography step using a Superdex75 column (GE Healthcare, USA) yielded highly purified KpDsbA, as judged by SDS-PAGE. Oxidized or reduced KpDsbA was prepared using a 25-fold molar excess of copper-(II)-1,10-phenanthroline or DTT, respectively. Oxidizing/reducing agent was then removed and the protein buffer-exchanged into 10 mM HEPES, pH 7.4 in one step using GE-25 Sephadex desalting resin for crystallization and biochemical experiments. 

Preparation of *E. coli* DsbA (CAA56736), *S. enterica Typhimurium* DsbA (AAB81592) and *E. coli* DsbC (AAA83074), lacking the periplasmic leader signal were purified as described for KpDsbA. For peptide oxidation experiments, *E. coli* DsbB (AAC74269) membrane extracts were prepared as described previously [19] and re-suspended in phosphate buffered saline (PBS, 137 mM NaCl, 2.7 mM KCl, Na_2_HPO_4_ 10 mM and KH_2_PO_4_, pH 7.4) containing 10 % glycerol.

### KpDsbA Complementation of EcDsbA

The ability of KpDsbA to rescue non-motile *E. coli dsbA* ^-^ null (JCB817) and *dsbA* ^-^/*dsbB* ^-^ double-null (JCB818) strains was assessed in a cell-swarming assay as described previously [16]. The mature KpDsbA coding sequence was cloned into pBAD33 under an arabinose inducible promotor with the EcDsbA periplasmic signal sequence. A wild-type EcDsbA cloned into pBAD33 vector was used as a positive control. Non-motile *E. coli dsbA* ^-^ deficient (JCB817) or *dsbA* ^-^ / *dsbB* ^-^ double-mutant (JCB818) [3] cells (2x10^6^) transformed with a KpDsbA or EcDsbA pBAD33 inducible vector were spotted onto the center of a soft M63 minimal agar plate containing 40 mg/mL of each amino acid (except L-cysteine). Plates were incubated at 37 °C and motility of cells monitored using a Molecular Imager® Gel Doc™ system from BIO-RAD (CA 94547, USA) after 3-7 h. Complementation experiments were repeated as biological triplicates. 

### KpDsbA Disulfide Reductase Activity

Under mild reducing conditions, DsbA proteins can reduce the intermolecular disulfide bonds formed between insulin chains A and B [3]. The rate of disulfide bond reduction can be spectroscopically followed at OD_650nm_ by an increase in turbidity resulting from production of the insoluble insulin chain B [20]. Samples were prepared in 1 cm cuvettes containing 10 μM of protein (KpDsbA, EcDsbA or EcDsbC), 0.33 mM DTT and 2 mM EDTA in 100 mM NaH_2_PO_4_ / Na_2_HPO_4_ titrated to pH 7.0. Catalysis was initiated by the addition of 0.131 mM insulin (I0516, Sigma-Aldrich, Australia) to the sample mixture. The assay was repeated three times and data were plotted showing standard deviations. 

### Measurement of KpDsbA Redox Potential

The standard redox potential of KpDsbA was measured using its intrinsic tryptophan fluorescence, as described previously for EcDsbA [6]. Oxidized KpDsbA was incubated for 12 h at 25 °C in degassed 100 mM NaH_2_PO_4_ / Na_2_HPO_4_ buffer (pH 7.0, 1 mM EDTA, 298K), containing 1 mM oxidized glutathione (GSSG) and varying concentrations of reduced glutathione (GSH) (0–2 mM). KpDsbA (200 µL) from each redox condition was dispensed into a 96-well plate (TPP AG, Switzerland #92096) and tryptophan fluorescence was measured (excitation at 280 nm, emission set to 332 nm) using a microplate reader (Synergy H1 and Gen5 2.0 software, Biotek, USA). Data were normalized and the redox potential was calculated as described for EcDsbA [6]. In brief, the equilibrium constant K_*eq*_ was calculated using the equation: Y = ([GSH]^2^ / [GSSH])/(K_*eq*_ + ([GSH]^2^ / [GSSH])), where Y is the fraction of reduced protein at equilibrium. The redox potential for KpDsbA was calculated from the Nernst equation: E^0’^_KpDsbA_ = E^0’^_*GSH/GSSH*_ - (RT/nF)lnK_*eq*_ where E^0’^_*GSH/GSSH*_ = - 240 mV, R is the ideal gas constant 8.314 JK^-1^mol^-1^, T is the absolute temperature in K, n is the number of electrons transferred (n = 2), F is the Faraday constant 9.648x104 Cmol^-1^ and K_*eq*_ is the equilibrium constant derived from the binding equation. All measurements were performed as biological triplicates. The graph shows a plot of the average values including error bars representing the standard deviation for the replicates. 

### KpDsbA Thiolate Anion pK_a_ Determination

The pH-dependent absorbance of the catalytic thiolate anion of KpDsbA was followed at 240 nm [21] using a CARY 50 UV/VIS spectrophotometer (Agilent Technologies, USA). The pH titration measurements of oxidized or reduced KpDsbA (40 μM) in 2 mL composite buffer (10 mM Tris, 10 mM sodium citrate, 10 mM K_2_HPO_4_, 10 mM KH_2_PO_4_, 200 mM KCl, and 1 mM EDTA) were conducted at 22 °C. Absorbance (λ = 240 and 280 nm) was measured between pH 6.5 and 2.0 in 0.25 increments. The pK_a_ value was calculated from the fitted curves of three replicates using the Henderson-Hasselbalch equation (pH = pK_a_ - log ([A240 ⁄A280]red ⁄ [A240 ⁄A280]oxid)). Experiments were repeated at least three times. Plotted data represent average values and error bars represent the standard deviations across the replicates.

### Relative Stability of Oxidized and Reduced Forms of DsbA Enzymes

Temperature-induced unfolding of native SeDsbA and KpDsbA was determined as described previously [13] using a Jasco J-810 circular dichroism (CD) spectropolarimeter (Jasco, USA). The redox state of the protein was confirmed using Ellman’s reagent [22]. The largest difference in molar ellipticity for oxidized or reduced enzymes was calculated from initial far-UV CD spectra (from 250 nm to 190 nm) recorded at 25 °C and 95 °C, respectively. The unfolding of oxidized and reduced protein (SeDsbA_ox_ = 220 nm, SeDsbA_red_ = 220.5 nm and KpDsbA_ox_ = 211 nm, KpDsbA_red_ = 209.5 nm) was monitored at a heat rate of 1 K / min from 298 K to 368 K in a 1 mm quartz cuvette. All measurements were carried out with 10 µM protein in 100 mM NaH_2_PO_4_ / Na_2_HPO_4_, 1 mM EDTA at pH 7.0. Samples for measurement of reduced enzyme contained 0.75 mM DTT. Raw data were analyzed in Prism and fitted to a two-state unfolding model as described previously [23]. The standard deviation was measured from three replicates.

### KpDsbA Dithiol Oxidation Activity

A peptide (CQQGFDGTQNSCK) with a 1,4,7,10-tetraazacyclododecane-1,4,7,10-tetraacetic acid (DOTA) group amide-coupled to the N-terminus, and a methylcoumarin amide-coupled to the ε-amino group of the C-terminal lysine, was purchased from AnaSpec (Fremont, CA). Lyophilized peptide was re-suspended in 100 mM imidazole, pH 6, at a concentration of 2 mM. Europium trifluoromethanesulfonate (Sigma Aldrich, Australia) solution (100 mM) was added to the peptide at a molar ratio of 2:1 and incubated for 5 min at room temperature, to allow europium chelation. The peptide solution was then immediately aliquoted, flash frozen in liquid nitrogen and stored at -80°C. An increase in fluorescence occurs upon oxidation of the peptide cysteines to form a disulfide. Thus, fluorescence can be used to monitor the capacity of DsbA enzymes to catalyse dithiol oxidation. 

Assays were conducted using a Synergy H1 multimode plate reader (BioTek, USA) with the excitation wavelength set to 340 nm and emission to 615 nm. A 150 μs delay before reading and 100 μs reading time were used for time-resolved fluorescence. The assay was performed in a white 384-well plate (Perkin Elmer OptiPlate-384, Part #: 6007290). The buffer consisted of 50 mM MES, 50 mM NaCl and 2 mM EDTA at pH 5.5. The reaction consisted of a 50 μL solution in each well, containing 160 nM EcDsbA, KpDsbA or SeDsbA, 1.6 μM EcDsbB (crude membrane extracts, containing ubiquinone) and 8 μM peptide substrate added last to initiate the reaction. Samples containing buffer and DsbA or buffer and peptide were used as controls. Data were measured for three replicates and are presented as mean values, with the standard error of the mean indicated by error bars. 

### KpDsbA Crystallization and Crystal Structure Determination

After initial screening using the UQ ROCX facilities, crystals of reduced KpDsbA were grown at 20 °C in VDXm 24-well plates (Hampton Research) using the hanging-drop vapor diffusion method. Screening plates were imaged and incubated in a RockImager 1000 (Formulatrix, MA, USA). Drops contained 0.5 μL of 180 mg/mL reduced KpDsbA and 0.5 μL of crystallization solution (0.1 M succinic acid pH 5.3, 25 % (w/v) polyethylene glycol 1500 and 15 % (v/v) 2-methyl-2,4-pentanediol). For diffraction data measurement, crystals were frozen in liquid nitrogen without additional cryo-protectant. Diffraction data were measured at the Australian Synchrotron micro-focus MX2 beamline using BlueIce software [24]. Reflections were processed in Mosflm [25] and XDS [26], analyzed and converted to MTZ in Pointless [27] and scaled in SCALA [27]. Phases were obtained by molecular replacement (MR) using PHASER [28] with EcDsbA as template (PDB code: 1DSB) . The initial model was improved by iterative model building in COOT [29] and refinement in PHENIX [30]. However, the progress of refinement was stalled with a high R-factor/Rfree of 25.7 % / 29.3 %. Diffraction data analysis in Phenix.xtriage indicated that the crystal was merohedrally twinned with a twinning fraction of 0.42. Further refinement cycles were performed using the twin target function as implemented in PHENIX with the twinning operator h,-h-k,-l. Two fold non-crystallographic symmetry (NCS) is present (which does not align with space group axes), though NCS was not used at any stage of refinement. The refinement finally converged after several TLS refinement cycles. No atoms were modeled into additionally spherical density located between chain D (L133) and chain B (T57) because it was not obvious what was bound. The stereochemical quality of the final model was assessed using MolProbity [31]. A summary of the data processing and refinement statistics are provided in Table 2. 

**Table 2:** X-ray data measurement and refinement statistics for KpDsbA.

**Data collection**	**Value**
Space group	P 32
Unit cell dimensions	
*a* (Å)	91.5
*b* (Å)	91.5
*c* (Å)	147.2
α, β, γ (°)	90, 90, 120
Wavelength (Å)	0.95369
Resolution (Å)	53.94 - 1.99 (2.10 - 1.99)
Number measured reflections	527,166
Number of unique reflections	94,694
R_merge_a	0.091 (0.566)
R_p.i.m._	0.043 (0.264)
<I>/<σI>	11.1 (2.9)
Redundancy	5.6 (5.5)
Completeness (%)	99.9 (99.9)
**Refinement statistics**	
Number of Reflections	94,693
Resolution (Å)	53.9-1.99 (2.02 -1.99)
R_free_ (%)	19.6 (31.9)
R_work_ (%)	16.1 (27.8)
Number of monomers in a.u.	6
Number of protein atoms	16622
Number of waters	371
*B* factors (Å^2^)	
Wilson	29.6
Protein atoms	39.4
Waters	41.4
RMSD Bond length (Å)	0.004
RMSD Bond angles (°)	0.740
Ramachandran favored / outlier (%)	97.4 / 0
Molprobity clashscore / score^b^	2.23 [99^th^(712)] / 1.12 [100^th^(12290)]

a The values in parentheses refer to the highest resolution shell.

b 100^th^ Molprobity [31] percentile is the best among structures of comparable resolution; 0th percentile is the worst. The number of structures included in the comparison is given in parentheses within square brackets.

Molecular figures were generated in PyMOL (The PyMOL Molecular Graphics System, Version 1.5.0.4 Schrödinger, LLC) and figures of the electrostatic potential were generated using APBS [32]. The surface, including the proportion of carbon atoms lining the hydrophobic groove in KpDsbA, was calculated using the CastP server [33], by averaging over all six molecules within the asymmetric unit. RMSD calculations and structural alignments were conducted using PyMOL as well as FATCAT [34].

### NMR Structure Determination of Oxidized KpDsbA

A sample of uniformly ^13^C,^15^N labeled oxidized KpDsbA (1.3 mM) was prepared in 50 mM MES (pH 6.5, 10% ^2^H_2_O and 90 % ^1^H_2_O). NMR experiments were conducted at 303 K on either 600 MHz or 800 MHz spectrometers equipped with cryogenically cooled probes. All spectra were acquired with standard pulse sequences and processed using TOPSPIN3.1 (Bruker BioSpin). H^N^, N, C^α^, C^α-1^, C^β^, C^β-1^ peak lists were generated manually in CARA using 2D [^15^N,^1^H]-HSQC, 3D HNCA, 3D CBCA(CO)NH and 3D HNCACB spectra and used as the input for automated backbone assignments using UNIO-MATCH. These assignments were refined manually and extended using 3D ^15^N-resolved [^1^H,^1^H]-NOESY. H^β^, H^α^ assignments were obtained using a 3D HBHA(CBCACO)NH spectrum. H^N^, N, C^α^ and C^β^ assignments together with H^β^, H^α^ were provided as input for UNIO-ATNOS/ASCAN for automated side-chain assignments using 3D ^15^N-, ^13^C_ali_ - and ^13^C_aro_ - resolved [^1^H,^1^H] NOESY datasets [35,36]. Upper limits for distance restraints used in structure calculations were automatically generated from NOESY datasets using UNIO-ATNOS/CANDID and the structure of oxidized KpDsbA was determined using the torsion angle dynamics program CYANA3.0 [37]. Conformers with lowest CYANA target function values were energy minimized using OPALp and validated using structure validation tools (http:/www.pdb.org/ and http:/www.nihserver.mbi.ucla.edu/). Structures were inspected and analyzed with MOLMOL [38]. Table 3 summarizes the NMR statistics. 

**Table 3:** Parameters for structure calculation and characterization of 20 lowest energy minimized NMR conformers of oxidised KpDsbA (1─188).

**Quantity** ^a^	**Value**
NOE upper distance limits	3859
intraresidual	813
short-range	1052
medium-range	969
long-range	1025
Residual target function value [Å^2^]	3.3 ± 0.2
Residual NOE violations	
number ≥ 0.1 Å	36.8 ± 7.5
maximum [Å]	0.16 ± 0.11
Residual dihedral angle violations	
number ≥ 2.5°	1.1 ± 0.6
maximum [°]	4.2 ± 3.2
AMBER energies [kcal/mol]	
total	-7513 ± 381
van der Waals	-562 ± 213
electrostatic	-8402 ± 159
**RMSD from mean coordinates^b^ [Å**]	
For well-defined regions (1-15,24-187)	
backbone	0.67 ± 0.17
heavy atoms	1.03 ± 0.13
For TRX domain (1-15, 24-62,146-187)	
backbone	0.55 ± 0.12
heavy atoms	0.99 ± 0.11
For helical domain (67-142)	
backbone	0.44 ± 0.08
heavy atoms	0.81 ± 0.09
**Ramachandran plot statistics^c^**	
most favoured regions [%]	77.7
additional allowed regions [%]	19.6
generously allowed regions [%]	1.2
disallowed regions [%]	1.5

^a^ Except for the top five entries (those relating to NOEs), average values and standard deviations for the 20 energy-minimized conformers are given. The top six entries represent the output generated in the final cycle of the UNIO-ATNOS/CANDID-CYANA3.0 calculation. ^b^ The numbers in parentheses indicate the residues for which the RMSD was calculated. ^c^ As determined by PROCHECK.

### Binding Affinity of DsbA-Interacting Peptides

Crystal structures of the EcDsbA:EcDsbB complex revealed that the P2 loop region of EcDsbB interacts with EcDsbA [39,40]. Two peptides derived from the P2 loop sequences of EcDsbB and KpDsbB (*Ec* – PSPFATCD and *Kp* – PSPFQTCD) were synthesized by solid-phase methods using Fmoc deprotection on rink-amide MBHA resin (leading to C-terminal amidation) and capped by N-terminal acetylation. Amidation and acetylation ensure that there are no charges on the peptide termini, as these are not present in the native DsbB loop sequence. Binding affinity was measured using a MicroCal™ Auto-iTC_200_ from (GE Healthcare, USA) at 25 °C. The sample cell was loaded with 200 μL of 100 μM KpDsbA or EcDsbA in 25 mM HEPES, 50 mM NaCl, pH 7.4, and DMSO 0.8 %. The peptide (3 mM) diluted in the same buffer was titrated with an initial injection of 0.5 μL into DsbA, followed by 19 consecutive injections (2.0 μL) offset by 180 s, while the solution was constantly stirred (1000 rpm). Data were fitted to a single-site binding model using MicroCal™ Origin 7.0 software (Origin 7 SR4 v7.0552). Experiments were conducted in triplicate and affinity and thermodynamic parameters are reported as means and standard deviations (Table 4).

**Table 4:** Affinity and enthalpy for DsbB-derived peptides binding to DsbA proteins^1^.

**DsbA**	**DsbB-peptide**	**Stoichiometry**	**K_d_ (μM)**	**ΔH (kcal/mol)**
EcDsbA	PSPFATCD	1.0	16.1 ± 1.8	-8.4 ± 0.1
	PSPFQTCD	0.99	10.9 ± 0.6	-9.1 ± 0.2
KpDsbA	PSPFATCD	0.93	17.9 ± 1.5	-9.5 ± 0.7
	PSPFQTCD	0.97	16.7 ± 0.6	-11.1 ± 0.2

1 Apparent dissociation constant (K_d_) and enthalpy of binding (ΔH) at 20 °C obtained from three independent ITC experiments. See Figure S3 for representative ITC traces.

### Comparative Sequence and Structural Analyses

The sequence conservation of ten virulence factors previously identified [2] as substrates of DsbA were analyzed here. Sequences from published and validated DsbA substrate virulence factors were taken from the original literature and used to search the publicly available UniProt database [41] for potential homologues in *E. coli*, *S.* enterica *Typhimurium* and *K. pneumoniae*. Most of the 10 factors were originally identified in those three organisms except YscC and Caf1M, which were initially reported in *Yersinia pestis*. A protein-protein BLAST search was performed using the UniProt bacterial genome database with a threshold of P < 0.0001. Unless stated otherwise, homologues were identified in pathogenic strains, i.e. *E. coli* UPEC O6:K15:H31 and EPEC O127:H6 / O55:H7, *S. enterica Typhimurium* SL1344 and non-motile *K. pneumonieae* (hvKP1 / MGH 78578 / NTUH-K2044). Sequence identity between homologues was extracted from the UniProt protein BLAST results. All other sequence alignments reported herein (e.g. for Table 1) were conducted using ClustalW2 [42]. 

## Results

### Binding Residues of EcDsbA are conserved in SeDsbA and DsbAs of Five Other *Enterobacteriaciae*

EcDsbA and SeDsbA share 86 % sequence identity and both have been characterized previously [14,43]. SeDsbA can complement EcDsbA [44] in a null mutant motility assay, indicating that SeDsbA is able to interact with the EcDsbA binding partner EcDsbB and with the EcDsbA substrate *E. coli* FlgI [45]. Both are weak disulfide reductants in the standard insulin reduction assay for redox enzymes [43]. Both are similarly oxidizing enzymes: the redox potentials of EcDsbA and SeDsbA are -122 and -126 mV, respectively [9,43], whereas the range for all DsbAs is -80 to -163 mV (Table **1**). In both EcDsbA and SeDsbA the measured pK_a_ of the nucleophilic cysteine is 3.3 [7,43], though values vary across all DsbAs from 3.0 to 5.1 (Table **1**). Although disulfide bonds generally stabilize folded proteins, the disulfide form of DsbA enzymes is destabilizing [6,7]. The melting temperatures of the oxidized and reduced forms of EcDsbA and SeDsbA are almost identical (reduced 350 K and 351 K; oxidized 341 K and 342 K, respectively) [13] (Figure **S1**), whereas the range of melting temperatures across all DsbAs varies considerably (Table **1**). Importantly, the crystal structures of EcDsbA and SeDsbA can be superimposed with an RMSD of 0.8 Å for 176 Cα atoms, whereas across all structurally characterized DsbAs the RMSD with EcDsbA varies from 1.3 Å to 2.9 Å (for 122-167 Cα atoms) (Table **1**) [14]. 

**Figure 1:**
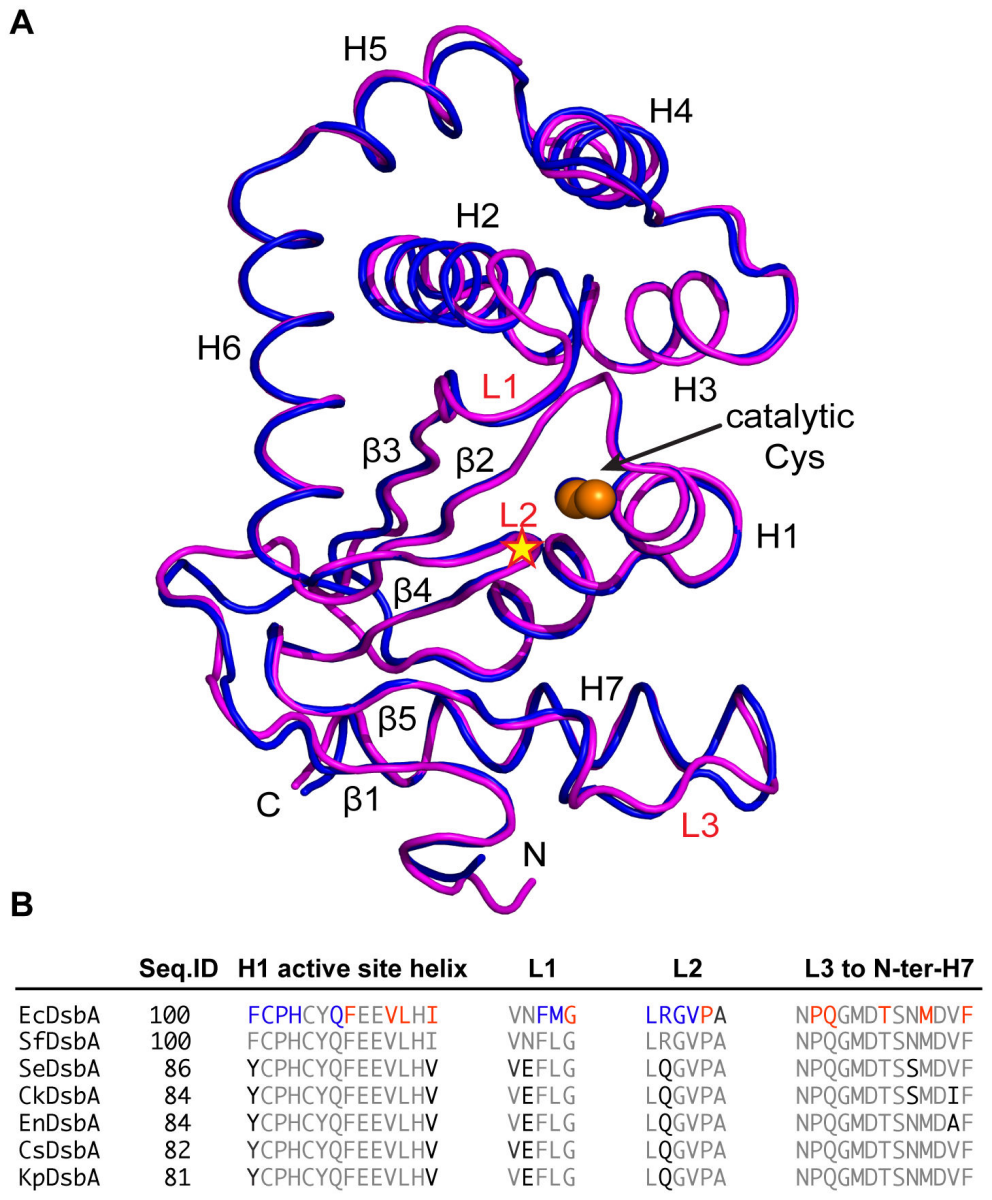
Comparison of EcDsbA and SeDsbA. **A**. Structural superposition of EcDsbA (magenta, PDB id: 1FVK) and SeDsbA (blue, PDB Id: 3L9S). N- and C-termini, helices (H1 - 7) and strands (β1-5) are indicated. In addition, surface loops (L1 – L3) predicted to be involved in binding EcDsbB periplasmic loop P2 or substrate are labeled in red. Active site cysteines are shown as orange spheres and the *cis*Pro motif in the L2 loop is indicated by a yellow star. **B**. Sequences of EcDsbA loops that bind DsbB (blue/red) or SigA substrate (blue). Homologues with highly conserved loop sequences are shown: *S. flexneri* (SfDsbA, P52235), *S. enterica Typhimurium* (SeDsbA E1WE53), *C. koseri* (CkDsbA, A8AL80), *E. cloacae* (EnDsbA, E3G5L9), *C. sakazakii* (CsDsbA, I2ED40) and *K. pneumoniae* (KpDsbA B5XZJ6). Conserved residues are shown in grey, and variable residues in black.

Two catalytically relevant EcDsbA complex structures have been described, a complex between EcDsbA and EcDsbB [39,40,46] and one between EcDsbA and a peptide segment of SigA, an autotransporter protein from *Shigella flexneri* [47]. Analysis of these structures revealed that the binding interface comprises the N-terminal regions of the active site helix H1, as well as loops L1 (the first of two loops connecting the thioredoxin and helical domains), L2 (the second of two loops connecting the thioredoxin and helical domains, also referred to as the *cis*Pro loop) and L3-H7 (residues in the loop preceding and at the N-terminal region of helix H7) (Figure **1A**). A hypothesis is that DsbAs sharing overall high sequence identity with EcDsbA and with highly conserved loop lengths and residues in these regions will share similar binding activities. As shown in Figure **1B**, SeDsbA falls into this cluster as does *Shigella flexneri* (SfDsbA, P52235), *Enterobacter cloacae* (EnDsbA. E3G5L9), *Citrobacter koseri* (CkDsbA, A8AL80) and *Cronobacter sakazakii* (CsDsbA, I2ED40) and *K. pneumoniae* (KpDsbA) (Figure **1B**). Of these, the DsbA with lowest sequence identity to EcDsbA is KpDsbA (81 %) encoded by an important human pathogen responsible for many antibiotic-resistant nosocomial infections [1,48,49]. To determine whether KpDsbA falls within the same class as EcDsbA, we investigated its structure, surface, redox and binding properties and compared them with EcDsbA. 

### KpDsbA Complements EcDsbA *in vivo*

The *E. coli* protein FlgI is required for *E. coli* motility and, in turn, FlgI requires the DSB machinery of *E. coli* to function. FlgI function is impaired in *E. coli dsbA* ^-^ deficient (JCB817) and *dsbA* ^-^/*dsbB* ^-^ double-mutant (JCB818) strains due to the absence of EcDsbA mediated dithiol oxidase activity [50]. As a consequence, these *E. coli* strains are non-motile. Intriguingly, *K. pneumoniae* is non-motile and does not encode a FlgI homologue. We tested whether KpDsbA was able to catalyse disulfide bond formation of *E. coli* FlgI using an *in vivo* complementation strategy [3]. We demonstrated that KpDsbA – like SeDsbA [44] – can fully restore the motility of *dsbA* ^-^ deficient strains, but not in the double *dsbA* ^-^/*dsbB* ^-^ mutant cells (Figure **S2**). This experiment shows that KpDsbA is able to oxidize FlgI cysteines and this requires the presence of EcDsbB.

Some distantly related DsbAs do not complement EcDsbA in this assay, including Gram-negative *Wolbachia pipientis* α-DsbA1 [23] and Gram-positive *Staphylococcus aureus* DsbA [13]. However, rescue or partial rescue of motility has been observed for a wide range of DsbA homologues, some sharing quite low sequence identity with EcDsbA, such as VcDsbA (40 %), PaDsbA (30 %) and NmDsbA1 (23 %) [15-17,51]. Consequently, EcDsbA complementation may not be a suitable guide for categorizing DsbA enzymes into distinct classes. 

### KpDsbA has redox properties almost identical to those of EcDsbA and SeDsbA

**Figure 2:**
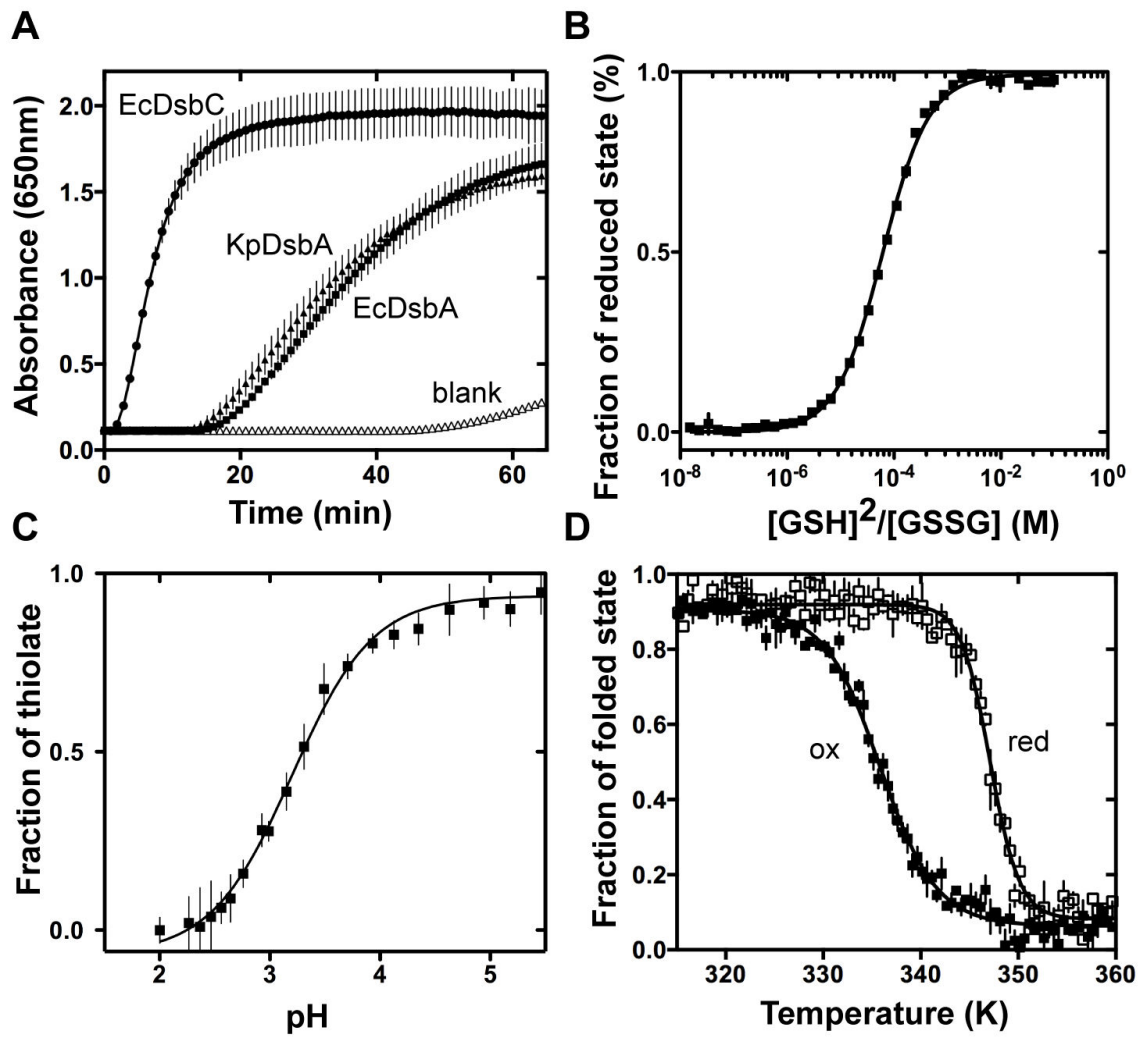
KpDsbA redox properties. **A**. Disulfide bond reduction activity of KpDsbA (▲), EcDsbA (■) EcDsbC (●) and a control without enzyme (△) was monitored spectrophotometrically. SeDsbA activity has been published elsewhere [43]. **B**. Redox equilibria of KpDsbA with glutathione (GSH/GSSG). **C**. Determination of the nucleophilic Cys33 (CXXC) pK_a_. The pH-dependent absorbance of the thiolate anion at 240 nm was fitted to the Henderson-Hasselbach equation **D**. Temperature induced unfolding of oxidized (ox, ■) and reduced (red, □) KpDsbA was determined by far-UV CD spectroscopy, showing that the reduced form is more stable than the oxidized form.

EcDsbA exhibits weak insulin reductase activity in the presence of dithiothreitol [52] whereas the *E. coli* disulfide isomerase EcDsbC is highly active in this assay. Reduction of the intermolecular disulfide bonds between the A and B chains of insulin results in precipitation of the B chain and this can be monitored by measuring the OD_650nm_. We found that purified recombinant KpDsbA has the same weak insulin reductase activity as EcDsbA (Figure **2A**) and SeDsbA [43]. The activity of other characterized DsbA enzymes varies. NmDsbA1, for example, has a much weaker activity than that of EcDsbA [15], and DsbA from *Mycobacterium tuberculosis* (MtbDsbA) is inactive in this assay [53]. In contrast, TcpG (VcDsbA) from *Vibrio cholerae* catalyses insulin reduction much faster than EcDsbA [54]. 

We next determined the standard redox potential of KpDsbA relative to glutathione ([GSH]^2^/GSSG, E^0’^ = -240 V). The equilibrium constant for KpDsbA was estimated from the [GSH]^2^/GSSG titration experiment to be 61.4 ± 0.1 µM (Figure **2B**), which corresponds to a standard redox potential of -116 mV. This value falls very close to the values reported for EcDsbA (-122 mV [9]) and SeDsbA (-126 mV [43]) considering the wide range of values reported across all DsbA enzymes (-80 to -163 mV) [14]. 

The pK_a_ value of the nucleophilic cysteine in the active site CXXC motif is a key determinant of DsbA reactivity towards substrate proteins. We measured the pK_a_ value for the nucleophilic cysteine of KpDsbA using pH-dependent thiolate absorbance at λ = 240 nm (Figure **2C**). The pK_a_^Cys30^ for KpDsbA was found to be 3.2, nearly identical to that of EcDsbA and SeDsbA (3.3) compared with the observed range for other DsbAs (3.0-5.1).

We also confirmed that reduced KpDsbA (T_m_^red^ 347.1 ± 0.2 K) is more stable than oxidized KpDsbA (T_m_^ox^ 335.8 ± 0.3 K) (Figure **2D**). The melting temperatures fall between values reported previously for EcDsbA (T_m_^red^ 350.9 ± 0.2 K, T_m_^ox^ 341.7 ± 0.2 K [7]) and those for SeDsbA (T_m_^red^ 351.2 ± 0.2 K, T_m_^ox^ 342.8 ± 0.4 K) reported here (Figure **S1**). Again, the range reported for all DsbAs is much wider (T_m_^red^ 337-357 / T_m_^ox^ 331-341 K) [51,54].

**Figure 3:**
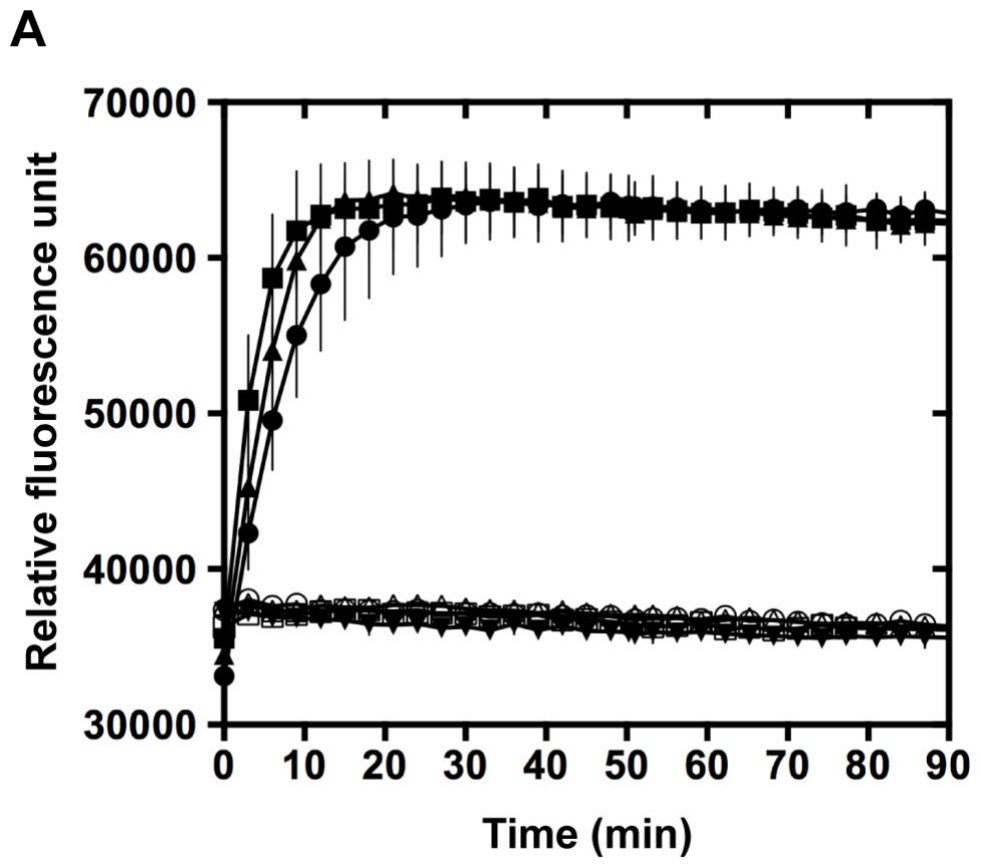
*In* *vitro* peptide dithiol oxidation. **A**. Dithiol oxidase activities of EcDsbA (■), SeDsbA (●) and KpDsbA (➉) were monitored using a fluorescently labeled peptide substrate. Samples lacking the partner protein EcDsbB (KpDsbA/peptide △), EcDsbA/peptide □, SeDsbA/peptide ○, or buffer alone ▼) showed no increase in signal over the same time period.

We then tested the dithiol oxidase activity of KpDsbA using a fluorescently labeled peptide substrate. The activity was monitored by the increase in europium fluorescence resulting from cyclization of the substrate peptide through formation of an intramolecular disulfide bond. In the presence of EcDsbB, we found that the rate for KpDsbA and SeDsbA catalyzed disulfide bond formation was almost indistinguishable from that of EcDsbA measured at the same concentration of enzyme (Figure **3**). This result suggests that KpDsbA (and SeDsbA) is able to interact in the same way as EcDsbA with the peptide substrate and with EcDsbB. TcpG has a similar activity to EcDsbA in this assay [54], whereas MtbDsbA is inactive in the presence of EcDsbB [53]. 

### Crystal structure of reduced KpDsbA

**Figure 4:**
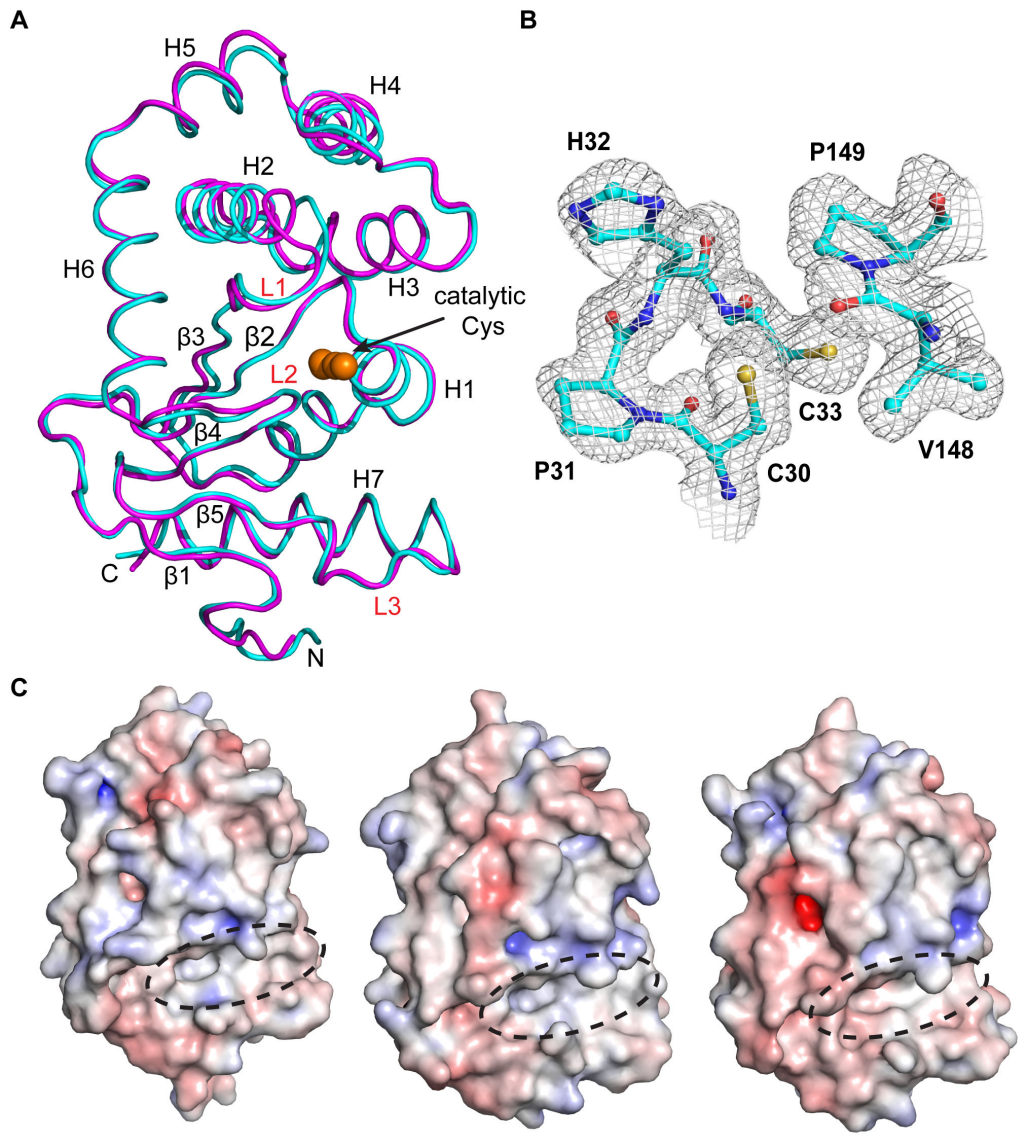
Crystal structure of KpDsbA. **A**. Superposition of crystal structures of KpDsbA (cyan, PDB Id: 4MCU) and EcDsbA (magenta, PDB id: 1FVK). The N- and C-termini, helices (H1 - 7) and strands (β1-5) are indicated. Surface loops L1 – L3 are labeled in red, and active site cysteines are shown as orange spheres. **B**. Electron density in the active site region of KpDsbA indicates that the cysteines are reduced. The 2Fo - Fc map was created using Phenix (model-map correlations) [30] and is contoured at 1.0 σ C. Electrostatic surface representation of EcDsbA, SeDsbA and KpDsbA (left, middle, right). Positive and negative electrostatic potentials are contoured from blue (+7.5 kT/e) to red (-7.5 kT/e). The hydrophobic grooves of all three enzymes are indicated by a dashed oval [8,43].

We determined the crystal structure of reduced KpDsbA (PDB: 4MCU) at 1.99 Å resolution by molecular replacement, using EcDsbA as the template. As expected, the structure is very similar to that of EcDsbA (Figure **4A**). The asymmetric unit contains six KpDsbA molecules each adopting the typical DsbA fold. Structural superposition of these six independent copies yielded a root mean square deviation (RMSD) < 0.45 Å for 176 Cα atoms between residues Gly6 - Val181. Likewise, structural alignment of KpDsbA with EcDsbA (1FVK, 1.7 Å, molecule B) and SeDsbA (3L9S, 1.6 Å) gave RMSD values < 0.9 Å for the identical range of 176 Cα atoms. By comparison, high resolution crystal structures of distantly related DsbAs have much higher RMSDs covering a smaller range of equivalent Cα atoms (e.g. PaDsbA (PDB code 3H93) and EcDsbA (1FVK, molecule B), 161 Cα atoms RMSD of 2.4 Å) [16]. These higher values are a consequence of structural deviations including a truncated helix H7 and a shortened hydrophobic groove. 

The structure of the catalytic site of KpDsbA is strictly conserved with that of EcDsbA, comprising the active site motif ^30^Cys-Pro-His-Cys^33^ located at the N-terminal end of helix H1 and the adjacent *cis*Pro (Val-Pro^151^) L2 loop (Figure **4B**). The cysteine residues (Cys30 and Cys33) are present in the reduced state in the crystal structure. A hydrophobic patch and a large groove surrounds the nucleophilic Cys30, as also occurs in EcDsbA and SeDsbA (Figure **4C**). As expected, these surface features are lined with residues contributed from the L1, L2 and L3 loops. 

**Figure 5:**
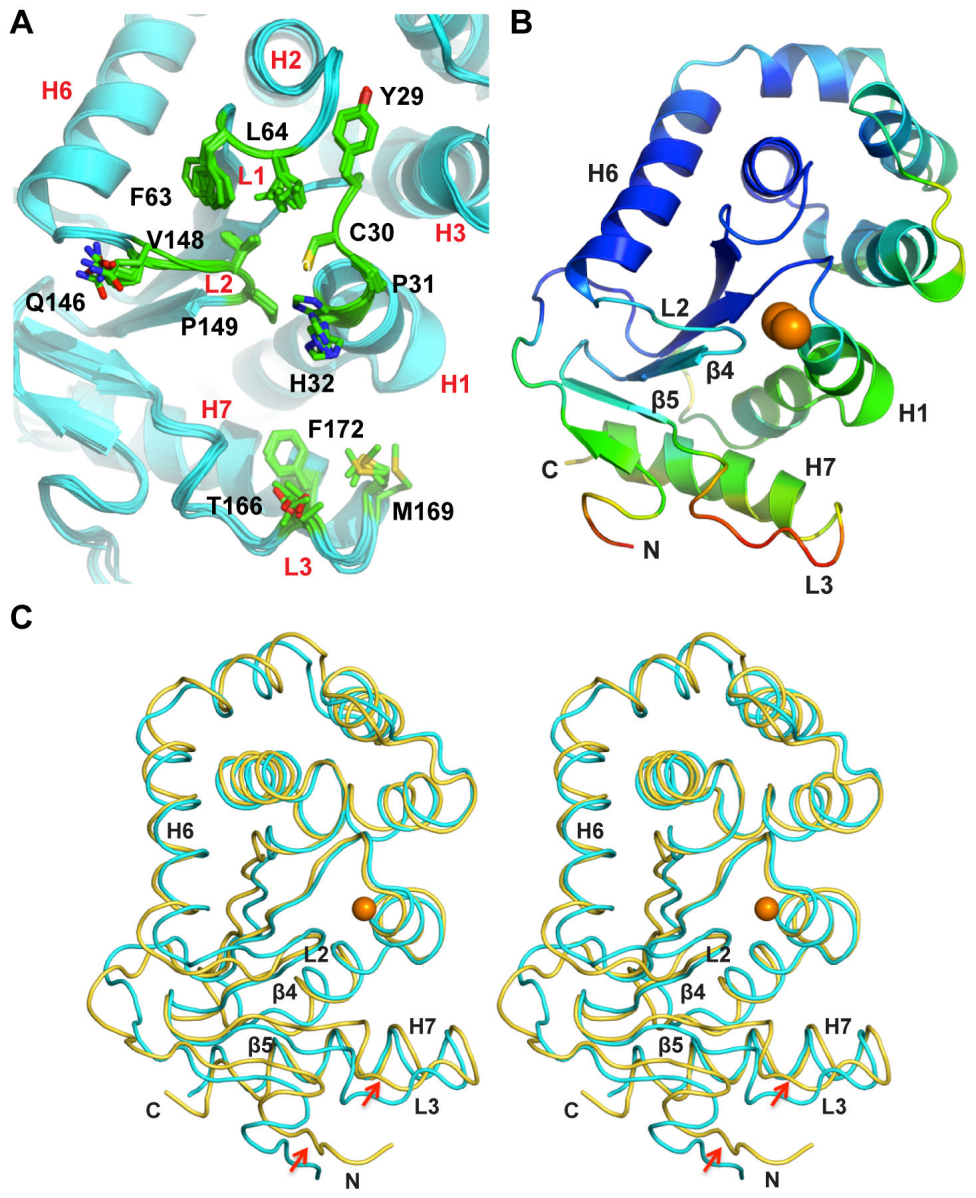
Conformational variability in X-ray and NMR structures of KpDsbA. **A** Superimposition of the six KpDsbA molecules (blue) in the asymmetric unit shows the limited conformational variability in the side chains of active site and L1, L2, and L3 loop residues (stick representation). **B**. Cartoon representation of the KpDsbA crystal structure (Molecule D), with C_α_ atoms colored by temperature factor (B-factor). Molecule D was selected as its temperature factor distribution is the most pronounced due to minimal crystallographic contacts. In particular, the high B-factor of loop L3 indicates mobility in that region, consistent with the NMR data **C**. Stereo diagram of representative states of reduced (X-ray, cyan) and oxidized (NMR, yellow) structures of KpDsbA. Red arrows highlight differences in the structures at N-terminal and L3 loop regions.

The six independent copies of KpDsbA in the crystal structure allow an analysis of conformational variability of the loop residues forming the binding surface. This revealed that the side chains of His32, Phe63, Leu64, Gln147, Thr167 and Met170 adopt various rotamer conformations, whereas there is no evidence of conformational variability in Tyr29, Cys30, Pro31, Val149, Pro150, and Phe173 (Figure **5A**). The side chain variations do not influence the surface accessibility of the hydrophobic groove, which was calculated to be 371 ± 32 Å^2^ by CastP [33] across the 6 molecules. Moreover, the hydrophobic nature of the groove is unaffected by the side chain conformational variability as indicated by the proportion of carbon atoms lining this groove (69 ± 3 %) [33].

### NMR Solution Structure of KpDsbA is Similar to the Crystal Structure

Previous studies have demonstrated that there are minimal differences between reported structures (crystal and NMR) of oxidized and reduced EcDsbA. To determine if this was also the case for KpDsbA, a semi-automated NMR approach was used to determine the structure of oxidized KpDsbA (PDB ID: 2MBS, BMRB ID: 19413). Following UNIO-ATNOS/ASCAN, manual verification and refinement enabled assignment of 89.2 % of the non-labile proton resonances in KpDsbA. These were used to generate the NOE-based distance constraints for final structure calculation. Twenty conformers with lowest target function and least violations of input restraints were chosen to represent the structure of oxidized KpDsbA (Figure **S4 A/B**). It was not possible to assign several backbone amide resonances corresponding to residues in the β1- β2 loop (Ile16, Gly18, Glu19, Gln21, Val22, Leu23), so that this region appears to be largely disordered in the NMR ensemble compared with the rest of the structure. The backbone (N, Cα, C’) and all-heavy atom RMSD for the 179 well-defined residues (1–15, 24-187) of the 20 KpDsbA conformers were 0.67 ± 0.17 Å and 1.03 ± 0.13 Å, respectively. Structural statistics are summarized in Table 3. As observed for other DsbA structures, the individual thioredoxin and helical domains can be superimposed with higher precision than the entire structure. This is most likely due to inter-domain motion, which has also been reported in the structures of EcDsbA [55] and VcDsbA [56]. Residues which fall into disallowed Ramachandran regions include the unassigned residues Glu19, Gln21, Val22, and His32, and residues in loop regions, i.e. Lys55, Phe63, Leu64, Asn155 and Met170. 

The overall conformation of the NMR structure of oxidized KpDsbA is similar to that of the crystal structure of reduced KpDsbA (Figure 5C). For example, superposition of molecule A in the crystal structure of reduced KpDsbA with the first structure in the NMR ensemble of oxidized KpDsbA, yields an RMSD of 1.09 Å over 169 Cα atoms. To make a similar comparison, the crystal structures of oxidized (1FVK, molecule B) and reduced (1A2L, molecule B) EcDsbA have an RMSD of 0.45 Å (over 186 Cα atoms) and the crystal structure of oxidized EcDsbA (1FVK, molecule B) and the first structure in the NMR ensemble of reduced EcDsbA (1A24) have an RMSD of 1.95 Å over 181 Cα atoms [57,58]. 

The structures of the catalytic sites and hydrophobic surface features are similar, considering that the cysteines of the CXXC motif are oxidized in the NMR structure and reduced in the crystal structure (Figure **S4C**). As has been noted previously for other DsbA solution and crystal structures [56,59], L3 of KpDsbA is a relatively flexible part of the protein in both NMR and crystal structures (Figure **5B and C**). Thus, overall the structures of oxidized and reduced KpDsbA are similar, notwithstanding the different conditions and approaches used for structure determination.

### Binding Affinity of DsbB peptides is similar for KpDsbA and EcDsbA

The similar surface features and similar predicted binding residues of KpDsbA and EcDsbA suggested that these enzymes would interact with binding partners with similar affinity. The crystal structures of the EcDsbA:EcDsbB complex showed that the second periplasmic loop P2 of EcDsbB binds directly to EcDsbA [39,40]. The binding residues are 98-PSPFATCD-104 and these are highly conserved in KpDsbB (98-PSPFQTCD-104). These two P2 peptides were synthesized and isothermal titration calorimetry (ITC) was used to assess their binding affinity for both enzymes. KpDsbA and EcDsbA were found to bind to PSPFATCD and PSPFQTCD with similar affinities (K_d_ 11-18 µM, Table **4**, Figure **S3A**). We investigated the interaction of KpDsbA with PSPFQTCD by structural superposition of KpDsbA onto the structure of EcDsbA in the EcDsbA:EcDsbB complex structure. Residue Ala of EcDsbB PSPFATCD was mutated *in silico* to PSPFQTCD, using the most commonly observed rotamer for glutamine. The superimposed model showed that the P2 loop matched the surface of KpDsbA very well, with no clashes apparent between the P2 residues and KpDsbA (Figure **S3B**). 

## Discussion

We have shown that the structural, surface, redox and binding properties of EcDsbA, SeDsbA and KpDsbA enzymes are highly conserved, and that these three DsbAs and four other DsbAs (from *Enterobacter cloacae*, *Citrobacter koseri*, *Shigella flexneri* and *Cronobacter sakazakii*) might be considered an *Enterobacteriaceae* subclass of DsbA. Carbapenem-resistant *Enterobacteriaceae* are responsible for a large proportion of difficult to treat community- and hospital-acquired infections [60] and there is an urgent need to develop novel therapeutic strategies to tackle these so-called ‘super bugs’ [61]. 

**Figure 6:**
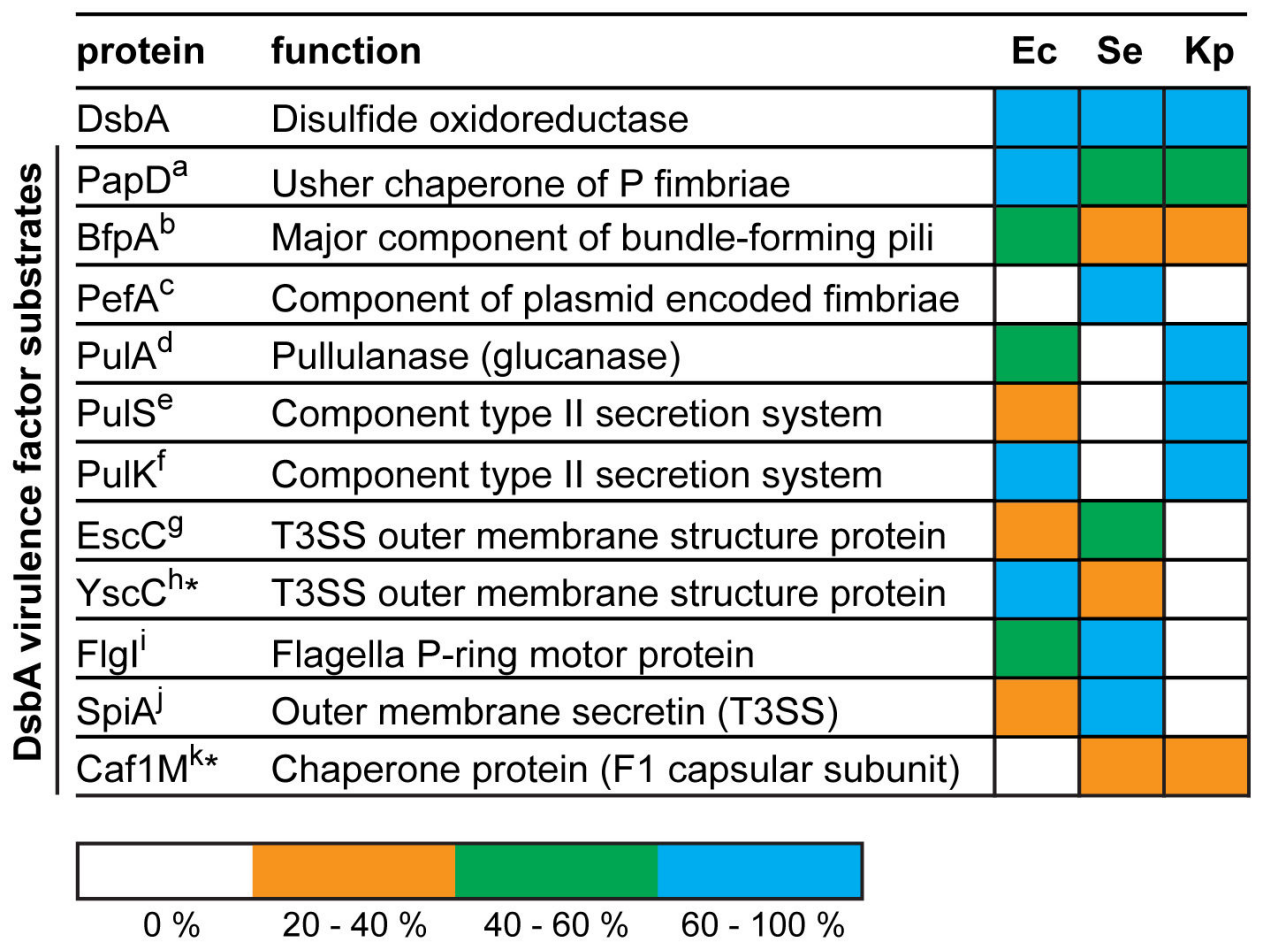
Conservation of DsbA substrate virulence factors. Comparison of the sequence conservation of DsbA oxidoreductases from *E. coli* (Ec), *S. enterica Typhimurium* (Se) and *K. pneumonia* (Kp) and of characterized DsbA substrate virulence factors. Sequence identities relative to the characterized substrate protein are represented in different colours, as shown in the key. White squares indicate the lack of a sequence homologue in the specific bacteria. ^*^YscC and Caf1M were identified as DsbA substrate proteins in *Yersinia pestis* [71,72]. ^a^ [68], ^b^ [73], ^c^ [44], ^d^ [74], ^e,f^ [75], ^g^ [5], ^h,k^ [71,72], ^i^ [45], ^j^ [69].

One approach to generate new classes of antibacterials is to target virulence rather than viability of bacteria. An antivirulence approach is predicted to lead to less selective pressure for resistance development, since most virulence traits are not essential for survival [62]. Targeting virulence may also expand the repertoire of antimicrobial targets, preserve the endogenous host microbiome and extend the lifespan of conventional antibiotics [61]. Most antivirulence strategies developed to date target individual virulence factors [61-65] and this has yielded some successes [66,67]. However, the armory of DsbA substrate virulence factors expressed in different *Enterobacteriaceae* varies (Figure **6**), so that drugs targeting specific virulence factors may not be effective against all *Enterobacteriaceae*. On the other hand, DsbA itself catalyzes assembly of many virulence factors [68-70] and DsbA knockouts severely attenuate virulence in infection models [12]. Targeting DsbA is therefore a compelling approach for the development of anti-virulence agents, because DsbA inhibitors should inhibit a range of virulence traits. Significantly, our findings point to the opportunity to develop a single antivirulence drug effective against DsbAs encoded by at least seven *Enterobacteriaceae* pathogens. 

The crystal structure and NMR solution structure of KpDsbA (the latter derived by semi-automated approaches) reported here are in excellent agreement. The availability of structural data for KpDsbA opens up the possibility of using structure-based approaches to generate DsbA inhibitors. Moreover, the close similarity of the crystal and NMR structures, and the use of semi-automated NMR, highlights how NMR can be used as an efficient first screen in e.g. drug-like fragment campaigns. By contrast, the six molecules in the asymmetric unit of KpDsbA crystal structure is far from ideal for rapid fragment-screening, but is nevertheless advantageous for follow up analysis.

Taken together, our data show that DsbA enzymes sharing >80% sequence identity with EcDsbA also share almost identical redox and surface properties and can thus be categorized as a distinct DsbA subclass. Further analyses will be required to determine how many subclasses of DsbA exist, and whether DsbAs with lower than 80% sequence identity will fall into the EcDsbA-like class. Importantly, our results suggest that compounds designed to inhibit EcDsbA will likely inhibit all DsbAs within the same class. Finally, we propose that compounds that bind KpDsbA might be identified rapidly using semi-automated NMR approaches, and that development of ‘hits’ to optimise potency can be achieved using a pipeline comprising biochemical and structural assays similar to those outlined herein.

## Supporting Information

Figure S1 **Thermal unfolding of SeDsbA.** **A**. Temperature-induced unfolding of oxidized (ox, ν) and reduced (red, θ) SeDsbA was monitored by far-UV CD spectroscopy. Unfolding was monitored in 1 K steps from 298 K to 368 K. Normalized average data points of three measurements were fitted to a two-state folding model. The reduced state of SeDsbA (351.2 +/- 0.2 K) is 9 K more stable than its oxidized (342.8 +/- 0.4 K) form.
(TIF)

Figure S2 **Summary of *invivo* complementation of KpDsbA and EcDsbA (**A**).** *E. coli* cells lacking *dsbA* ^*-*^ (JCB817) or *dsbA* ^*-*^ */dsbB* ^*-*^ (JCB818) are non-motile. Expression of KpDsbA or EcDsbA can rescue the swarming of *E. coli* *dsbA* ^*-*^ (JCB817) but not of *dsbA* ^*-*^ */dsbB* ^*-*^ cells. Expression of KpDsbA or EcDsbA is induced by inclusion of arabinose (arab).
(TIF)

Figure S3 **Binding studies of PSPFQTCD to KpDsbA.** **A**. Representative ITC profile for PSPFQTCD peptide binding to EcDsbA. For all combinations tested see Table 4. **B**. Model of the interaction of the KpDsbA (molecule A) with PSPFQTCD generated by structural superposition on the EcDsbA:EcDsbB complex [76].
(TIF)

Figure S4 **NMR structure of oxidized KpDsbA.** **A**. Overlay of the 20 NMR models; disordered region highlighted in blue. **B**. lowest energy NMR conformer. c. magnification of the active site region showing the disulfide bond formed between the cysteines in the averaged NMR solution structure of oxidized KpDsbA.
(TIF)
