# Supplementary figures and images for: Comparative Sequence, Structure and Redox Analyses of Klebsiella pneumoniae DsbA Show That Anti-Virulence Target DsbA Enzymes Fall into Distinct Classes
Source: PLoS One. 2013 Nov 14;8(11):e80210. doi: 10.1371/journal.pone.0080210 (PMC3828196; doi:10.1371/journal.pone.0080210)

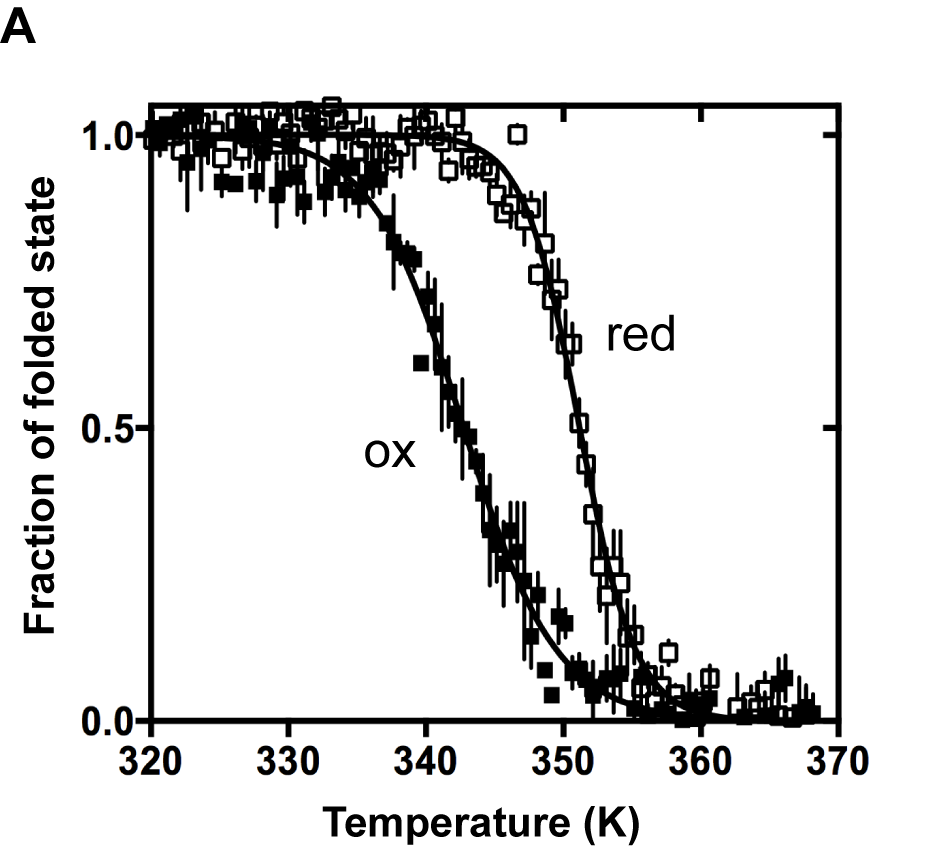

Supplement: Figure S1 — Thermal unfolding of SeDsbA. A. Temperature-induced unfolding of oxidized (ox, ν) and reduced (red, θ) SeDsbA was monitored by far-UV CD spectroscopy. Unfolding was monitored in 1 K steps from 298 K to 368 K. Normalized average data points of three measurements were fitted to a two-state folding model. The reduced state of SeDsbA (351.2 +/- 0.2 K) is 9 K more stable than its oxidized (342.8 +/- 0.4 K) form. (TIF) [file pone.0080210.s001.tif]

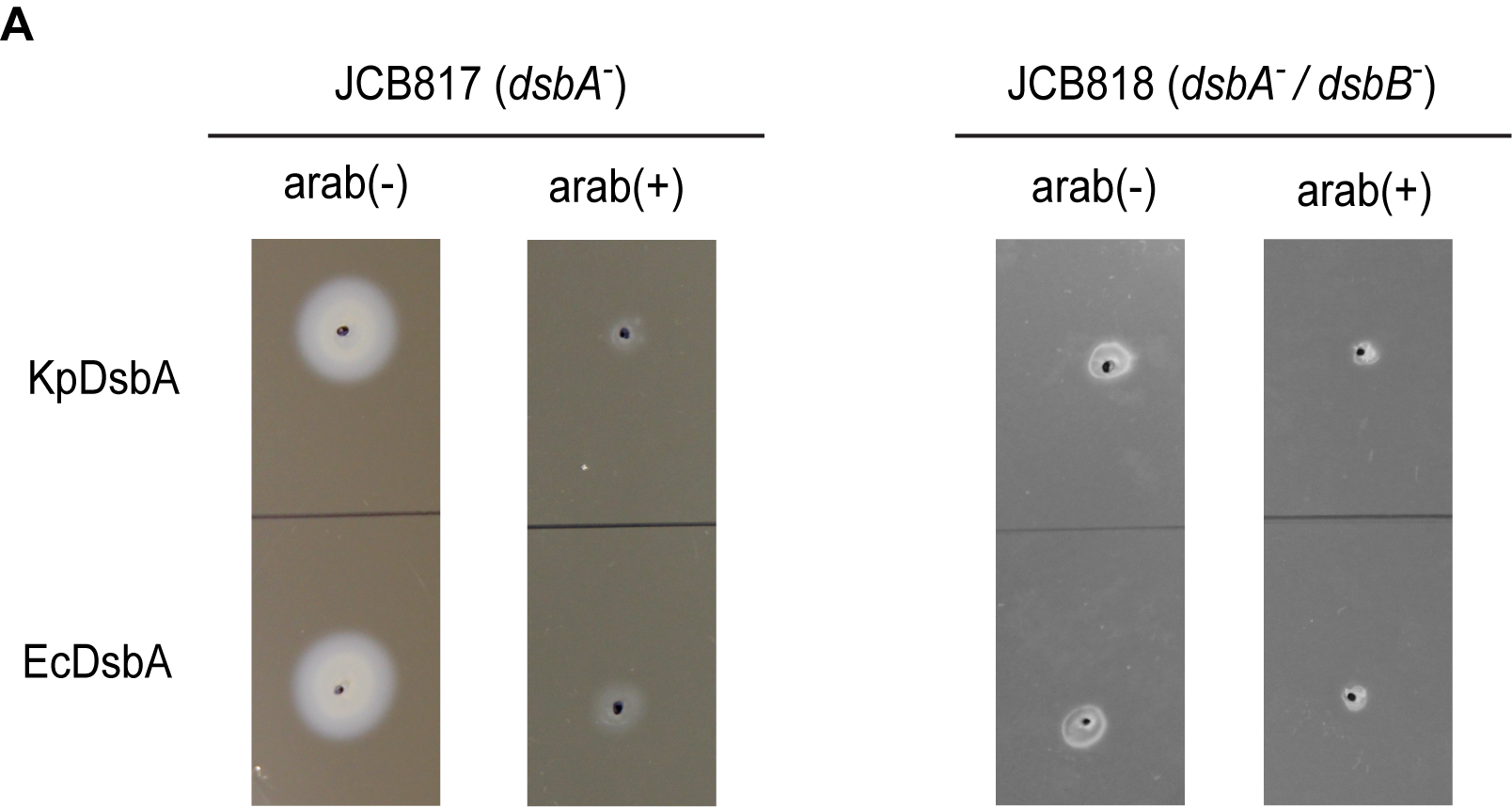

Supplement: Figure S2 — Summary of invivo complementation of KpDsbA and EcDsbA (A). E. coli cells lacking dsbA - (JCB817) or dsbA - /dsbB - (JCB818) are non-motile. Expression of KpDsbA or EcDsbA can rescue the swarming of E. coli dsbA - (JCB817) but not of dsbA - /dsbB - cells. Expression of KpDsbA or EcDsbA is induced by inclusion of arabinose (arab). (TIF) [file pone.0080210.s002.tif]

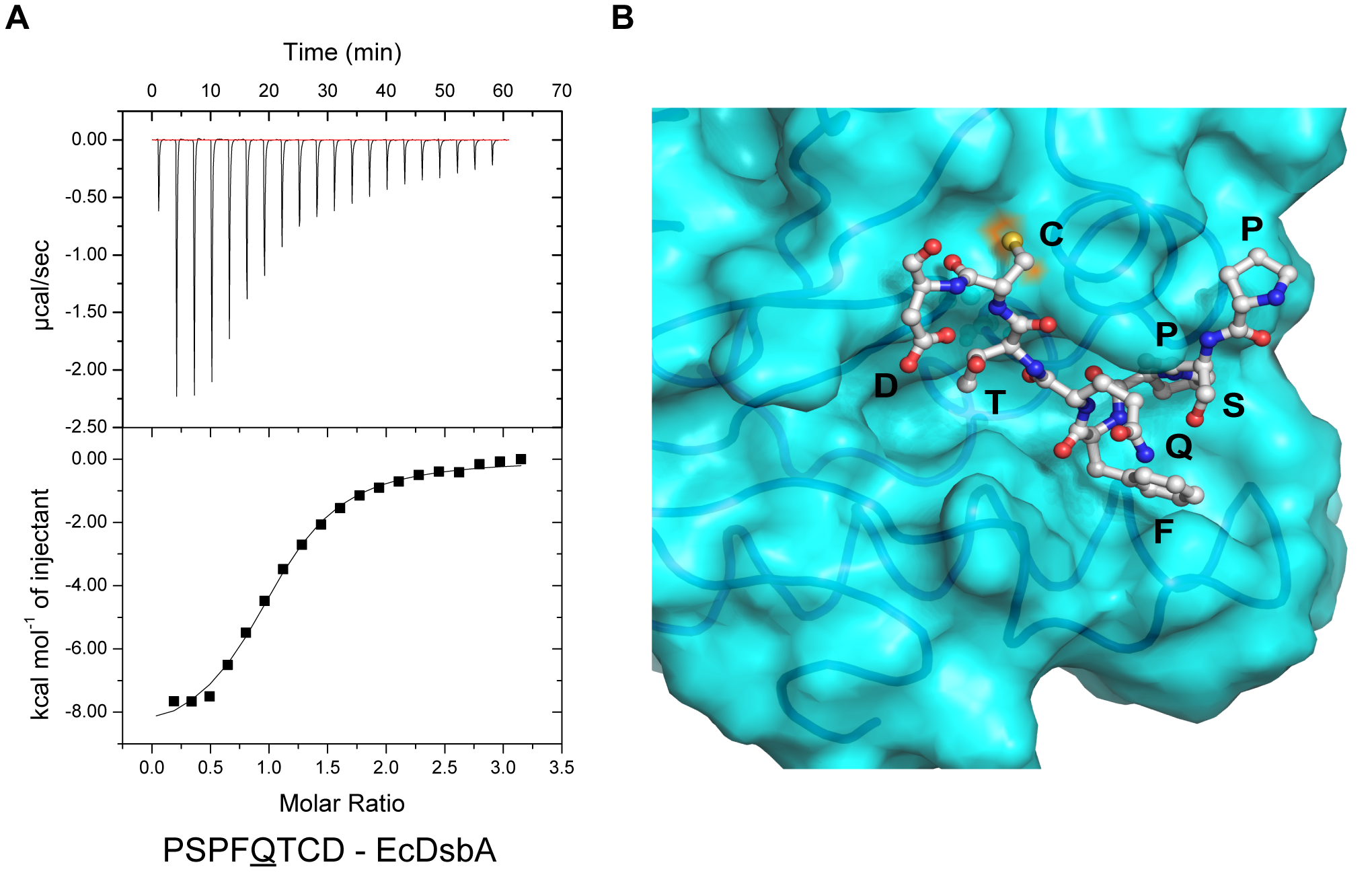

Supplement: Figure S3 — Binding studies of PSPFQTCD to KpDsbA. A. Representative ITC profile for PSPFQTCD peptide binding to EcDsbA. For all combinations tested see Table 4. B. Model of the interaction of the KpDsbA (molecule A) with PSPFQTCD generated by structural superposition on the EcDsbA:EcDsbB complex [76]. (TIF) [file pone.0080210.s003.tif]

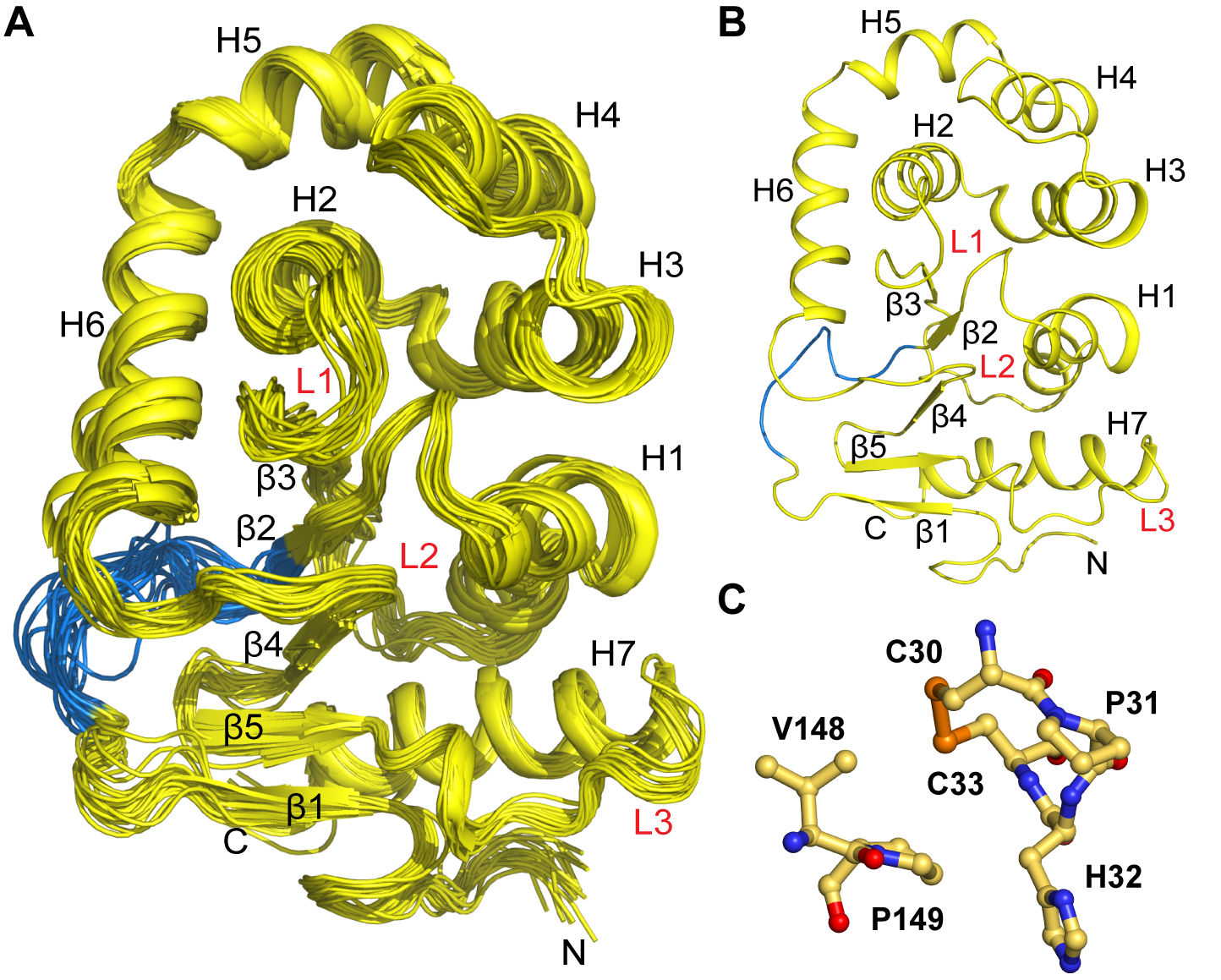

Supplement: Figure S4 — NMR structure of oxidized KpDsbA. A. Overlay of the 20 NMR models; disordered region highlighted in blue. B. lowest energy NMR conformer. c. magnification of the active site region showing the disulfide bond formed between the cysteines in the averaged NMR solution structure of oxidized KpDsbA. (TIF) [file pone.0080210.s004.tif]
